# Ten simple rules for teaching applied programming in an authentic and immersive online environment

**DOI:** 10.1371/journal.pcbi.1009184

**Published:** 2021-08-05

**Authors:** Frances Hooley, Peter J. Freeman, Angela C. Davies

**Affiliations:** School of Health Sciences, The University of Manchester, Manchester, United Kingdom; Carnegie Mellon University, UNITED STATES

## Introduction

Clinical bioinformatics is an applied discipline combining computer science with genomics in clinical practice. With the widespread integration of genomics into healthcare, trained clinical bioinformaticians are in short supply, but high demand, necessitating creative and flexibly-delivered education to fill the skills gap [1]. Since 2013, The University of Manchester has taught a masters programme to train clinical bioinformaticians working in the United Kingdom National Health Service (NHS). Following the success of this programme, in 2019, we expanded our portfolio and launched a fully online Postgraduate Certificate in Clinical Bioinformatics [2], designed to teach healthcare professionals and those with an interest in clinical bioinformatics, using authentic genomic clinical case studies and real-world examples. This course has now run for 2 cohorts with 45 students to date.

The Introduction to Programming unit was designed to provide a foundation course for those learning to code within the discipline of clinical bioinformatics. As this field is relatively new, those taking the course from the 2 cohorts came from a variety of backgrounds with diverse levels of experience in programming, ranging from novice programmers to those with professional roles in programming, from non-healthcare disciplines. Importantly, these students represented a global cohort, separated by diverse time zones, including UK, Saudi Arabia, and Australia. This course has also been provided for an additional 11 students on the Scientist Training Programme (STP) [3], training to be clinical bioinformaticians within the NHS, during emergency transition to remote teaching in 2020 to 2021; therefore, it needed to provide a diverse level of support to encourage all students to develop their programming skills. It also needed to be delivered entirely online in a mostly asynchronous way to support students from different global time zones who were also juggling work, family, and study commitments. It was therefore essential to find ways to build and maintain engagement that didn’t rely on synchronously delivered webinar-based communication [4]. Following these initial runs of the course, we would like to share 10 simple rules based on our experiences from delivering an applied practical course to a diverse set of students in a flexible, supportive, and engaging online learning environment.

### Introducing the 10 rules

The 10 rules are grouped into 3 themes: teaching, applied practice, and the team, as illustrated within Table 1. The first theme, teaching, incorporates the rules that involve understanding your student cohort (well), defining your pedagogy, and incorporating authentic tools, accordingly. The second theme, applied practice, relates to designing the content in relation to real-world problems that the students would likely encounter, in this case, in clinical practice. The third theme, the team, describes the benefits of embracing a multidisciplinary teaching team and a well-facilitated learning journey.

**Table 1:** Summary of themes for 10 rules.

Theme	No.	Rule	Description
TEACHING	1	Know your Students	This is an essential rule and applies to all teaching projects. However, it is particularly pertinent for this course where we have had a diverse set of student needs and levels of experience to support. By knowing students’ needs prior to delivering the course, educators can preempt topics and tailor support accordingly.
	2	Follow a pedagogy-first approach	In technology-enhanced learning projects, the pedagogy can be overshadowed by the technology. This can be even more of an issue when the tools themselves are core subject matter of the course. By focusing on the teaching methods and ignoring the software and platforms at the start, educators can build the right foundations to ensure all the learning objectives are embedded effectively in the students’ learning journey.
	3	Incorporate commonly used tools	Fundamental to the learning design was that the tools included in the course needed to be actively used by clinical bioinformaticians in practice. By adhering to an authentic and practice-driven experience during the course, the students are equipped to develop their skills post-course with the experience, insight, and confidence to be effective from day one.
	4	Create coding snippets to scaffold the learning	The Jupyter Notebooks developed for the course contained short blocks of coding accompanied by instructional content that helped build the learning incrementally. By incorporating self-directed and interactive materials in a simulated safe environment, the students can practice, experiment, and hone their programming skills [5]. This safe-to-fail learning space is particularly critical for clinical bioinformaticians whose role is to apply their programming skills to genomic data to help inform patient diagnosis and care.
	5	Design with flexibility in mind	Teaching in the moment to ensure that learning goals are met and students are supported was a really important rule learnt from running this course. It was made possible from the robust design methods and trusted pedagogic frameworks the course was built on. By pivoting to support students during delivery, we could meet their needs during the remote emergency teaching of the pandemic. It is also a lesson that we will apply to our teaching in general.
APPLIED PRACTICE	6	Bring it back to practice	Projects for the final coding assessment included work on real-world tools, such as VariantValidator [6]. By directly improving tools used by clinical bioinformaticians, the students could see the impact of their work, thereby increasing their motivation during and after the course.
	7	Build in real-world problems and methods	The students provided their own programming challenges, which they worked on in the team activities. By sourcing real problems to tackle, the students were being prepared for professional practice. By incorporating these within a simulated environment, they were learning to apply their skills using the tools and methods used in practice.
	8	Simulate a community of practice (CoP)	Clinical bioinformatics, like other professions, could be made more efficient through a strong collaborative network to streamline and share the development of resources used in practice. By equipping students with the necessary skills to develop their networks and to learn to work collaboratively from the beginning, this can occur after the course.
THE TEAM	9	Try a team-teaching approach	Having a course team from mixed disciplines and backgrounds sparked various innovative approaches in this course. This is a rule that has transferred across various different courses and has worked well for the team.
	10	Facilitate the journey … well!	Good facilitation is essential in all online delivery, so this rule is a fundamental one for online learning. This course recruited facilitators from the student community, provided pedagogic guidance, and ensured the facilitators were valued members of the team, which translated to the community feel of the course and encouraged engagement.

### Rule 1: Know your students

Knowing student preferences, requirements, and motivations has been a guiding principle for technology-enhanced learning projects for some time [7]. It’s important because it can help design the right scaffolding of activities to support high-order learning and inform techniques during delivery, which encourage engagement and build a sense of community [8]. We strongly believe that this is the most important rule, as it also began with user-centred design and cocreation of materials that informed our decision-making throughout the course development. Throughout the evolution of our clinical bioinformatics education at The University of Manchester, we have embedded an ethos of collaboration and cocreation with our students, driven by the ever-changing needs of a rapidly evolving national and international genomic medicine service [9]. When designing a course unit, we believe that it is best practice to gather learner requirements to define their actual needs before entering the design phase. This will help to hone the course content and focus the learning journey into a “critical path” of learning. We found that this resulted in asking lots of seemingly mundane questions like, “Is it essential, do they really need to know this?”, “What would a Clinical Bioinformatician do in practice?”, and “Does this really support those new to programming?”, which enabled us to maintain the unit’s focus on applied programming rather than theoretical programming.

To validate our approach, we ran a poll just before the course started to ask our students to consider how they felt about programming and to reflect on what they wanted to learn. The results reinforced our findings during the design phase, confirming that our students were generally novices but with some who had come from a more coding background that felt more advanced in their skills. Table 2 below shows the mix of results from a poll at the start of each of the courses asking the students their level of knowledge in programming.

**Table 2:** Results of poll investigating experience of programming.

Course:	PG Cert Run 1	PG Cert Run 2	STP Run 1	Totals
Number of students:	30	36	12	
I’ve never done it	13	13	2	28
I have dabbled with the basics in the past	6	8	1	15
I’ve done some in the past at a basic level	2	10	7	19
I regularly code but would see myself as intermediate	1	1	2	4
It’s essential in my job and would consider myself advanced	8	4	0	12

### Rule 2: Follow a pedagogy-first approach

The clinical bioinformatician’s environment is ever-changing, with new technologies developing at speed, the pipelines that they develop need to be regularly and rapidly updated, validated, and verified to be used clinically. The move towards reuse and sharing of code and working in an agile manner across different hospitals will enable clinical bioinformaticians to keep pace with these requirements. As such, the development of softer skills, including team working, and customer-driven software development were as fundamental as the core programming skills. We chose a pedagogy-first approach to the learning design, which meant prioritising the teaching methods over technology. By doing this, we could focus on “how” the programming activities and interaction with tools and other students could be scaffolded into the learning journey in order to meet the learning objectives [10]. It is very tempting to start with the tools, websites, and software the students will need, when designing a course developing programming skills. However, by focusing on the student learning needs and the course objectives, we could clearly define 3 pedagogic themes to design the course.

#### Social learning

In a fully online course, learning can take place by interactions between learner and content, learner and educator, and also between learners. Using online discussion fora blended with social media and carefully scaffolded real-world problems and questions, the online learning environment can be used to create and support interactions and develop learning between students. We embedded so-called social constructivist learning within our course design to encourage the students to learn collaboratively and build on and share their existing knowledge. Primarily, we did this to embed a supportive and collaborative learning environment. The continual flow of discussion in social learning, which involves the articulation and exchange of ideas, drew us to Laurillard’s Conversational Framework [11] as a pedagogic model. Laurillard’s framework centres the student at the heart of their journey and looks at learning as an iterative process that ebbs and flows as it grows. To put it plainly, the students would gradually build on their own knowledge through interaction with others and from internal reflection. This helped the team to design activities that encouraged peer-supported learning that not only helped the students but also helped us to be agile during delivery and focus our support on those students who needed it the most.

#### Problem solving and self-directed learning

The types of problems we embedded were designed to reflect the real-world challenges a clinical bioinformatician would encounter. It was essential to develop the problem-solving skills in a practical programming course, not only during the course but to instil self-regulated learning skills in order to apply the programming tools and techniques after the course had ended [12].

#### Situated learning

We wanted to simulate real-world practices to situate the students in how they will apply their programming skills. This included the potential challenges they may face in a volatile clinical environment and, importantly, to develop the skills and knowledge to overcome these challenges in a real-world setting. The provision of a realistic programming environment was more important than the rigour of the actual coding skills in this course, skills that would be developed during application in practice.

### Rule 3: Incorporate commonly used tools

Common and industry-standard tools that clinical bioinformaticians use within practice will include Slack, including dedicated channels to specific aspects of coding and GitHub, which is used as a repository to develop and share code using version control. We integrated these key tools into the course design to simulate a real-world programming environment as described below.

#### Social media

Students can often experience feelings of isolation commonly associated with distance learning [13]; therefore, it is essential within a social constructivist approach to integrate good discussion-based tools into the pedagogic model. The platform we chose was Slack. Slack is a collaboration tool for communicating in teams and consists of a workspace with individual channels that can be shared in the teams or made private [14]. General discussions, team strategy, and product owner interaction (where an educator would act as the customer for the coding task, providing user requirements and feedback on code iterations) were delivered through Slack. It was also used for group work and educational support, such as solving initial configuration issues with, e.g., Jupyter Notebooks and Python environments, pastoral support, and providing personal feedback on activities. The students demonstrated great team-working skills within groups of approximately 5 members providing peer-to-peer support and lessening isolation. This peer-supported learning involved over 10,000 messages and approximately 130 resources shared across the 3 cohorts of 78 students. Slack was used to provide real-time support on the coding sprints; this was a lightweight, but easily accessible means by which students could highlight problems encountered and get rapid feedback from their peers, facilitators, or course tutors.

#### GitHub

The course content was delivered outside of Blackboard (learning management system) to the students using GitHub [15]. GitHub is an industry-standard code hosting, version control, and collaboration platform and is suited to this unit because it lets users work together on projects from anywhere in the world. Initial introductory materials were delivered through Blackboard. Students were taught how to configure their own machines for programming using Anaconda to install Python3, and Windows users installed and initialised a LINUX environment, e.g., git bash, so that all students could be taught using LINUX. Students also installed git so that the course material could be downloaded from GitHub. Course material was taught using Jupyter Notebooks downloaded from GitHub [16]. The reason for this was to give the students an opportunity to get to grips with a commonly used version controlling tool they would encounter in practice. The notebooks were held in a GitHub private repository, which the students accessed as collaborators so they could then download them locally to work on their own parts of the project, as a bioinformatician would do. We also included a wiki in Git to collate any useful tools and Git Issues in the workflow of the tasks to collate any issues with the coding. We encouraged the use of community platforms and resources commonly used such as Stack Overflow [17] in the group work. The use of these tools and agile methods meant that they were experiencing the working practices of a clinical bioinformatician. Importantly, however, they were doing this in a way that would help to build their confidence, encourage them to learn from each other, build their professional network, and work with the toolkit they would use after the course had ended.

### Rule 4: Create coding snippets to scaffold the learning

In order to deliver the learning materials in an engaging and immersive way, we used Jupyter Notebooks. These are browser-based interactive notebooks that have editable code next to the instructional teaching content. These have been mainly used in research to support the reproducibility of code [18]; however, the ability to juxtapose practical and instructional content really lends itself to teaching. The format of the Notebooks allows for rich content to be created in order to interact with the code and data contained in such a notebook to form an educational narrative; we have integrated these throughout our health informatics teaching [16]. This is because the blend of activities can be mixed in a variety of ways to meet the learning objectives, for example, they can include relevant problems for students to solve interspersed with informative content and working examples all of which help to build learning incrementally. We created 12 Jupyter Notebooks that contained exercises with executable code examples in short snippets alongside practice-based tasks. These helped students practice coding in a fail-safe environment while learning at a pace that suited them.

### Rule 5: Design with flexibility in mind

Early investment in planning the learning journey from a learner-centric perspective will be time well spent. To plan this unit, we used the ABC Learning design toolkit, which involves the whole teaching team in an interactive 90-minute planning session that incorporates the 6 learning activities within Laurillard’s framework. It results in a basic storyboard, providing detail of the learning objectives, the indicative content, how it will be delivered (video, quiz, discussion), the time that each learning activity will take, and who will own each piece of content creation [11].

We found storyboarding the learning journey essential in order to create a flow or narrative in this unit. Using this approach, individual activities could be created that fitted well into the context of the entire unit. It also provided a great way of project managing the development of the unit including progress checking, identification of risks and issues, and providing ownership to content authors. The use of Kanban-style project management tools such as Trello boards to create the storyboard and assign activities works well if you are working in a large development team, or there are always excel sheets as an adequate alternative.

Independent of the tool used, we would recommend you have some way of tagging the learning activities so you see their spread and mix and ensure your chosen methods are permeating through your materials. For example, if you are trying to build a social or situated learning experience, then you don’t have a sequence of passive activities such as reading or watching a video. The storyboard can be continually reviewed to ensure that passive activities are interspersed with more investigative, collaborative, or production-oriented activities. This storyboard of activities also helped us to flex the learning journey to meet the different students’ needs during delivery of the course. For example, during the STP course unit, we changed the order and amount of time on tutorials that weren’t needed (such as on shell scripting) and spent more time on requested activities (such as GitHub tutorials) and changed the timetable to spread the learning across a longer time period. This agile teaching approach was achievable because of the robust instructional design methods during the storyboarding phase.

### Rule 6: Bring it back to practice

Students are more likely to be invested in the code they create if they can see that it will make a real difference to the delivery of genomic medicine. Therefore, the coding requirements were driven by and designed to meet the brief of real-world genomics problems sourced from direct interaction with clinical genetic scientists and were focussed on creating code to develop VariantValidator. VariantValidator is a popular Open Source tool that helps scientists to accurately describe genetic sequence variants using approved clinical nomenclature [6]. Aspects of the code created by the students have been tidied up, documented, and integrated into the VariantValidator. In addition, some students have been involved in ongoing work on the community-focussed VariantValidator project where their code will make a real difference in genomic medicine. Students have the opportunity to learn how to maintain and evolve code in a live development environment with expert guidance provided by the VariantValidator product owners and administrators. Using this model, the students could see that their work, once it reaches a certain standard, added to the clinical bioinformaticians toolkit via the VariantValidator utilities. This motivated the students hugely and is something that is now being developed for future cohorts to encourage engagement and to ensure the relevance and currency of the course. It also encourages collaborative development and validation of a central resource that can streamline healthcare practice, whereby healthcare scientists develop a single tool that meets all their needs rather than locally focussed tools that meet the needs of a single hospital. Such efficiencies will be essential in light of stretched healthcare budgets following the COVID-19 pandemic.

### Rule 7: Build in real-world problems and methods

We simulated real-world experience with the tools, platforms, and methods to provide a situated learning environment to encourage the use of current best practices for programming in clinical bioinformatics. Clinical bioinformaticians, like many other software developers, will not work in isolation but will work in teams, implementing agile software development, generating pipelines and software interfaces in collaboration with other members of the genetic laboratories.

We drew on agile software development methodologies by delivering the team coding projects in sprints, which dealt with real-world genomics issues while developing their programming skills. Sprints are short activities in a specified timeframe, which involve team members working collaboratively on a defined goal provided by a customer or User Story [19]. They have distinct phases starting with planning, working (or coding), reviewing, and reflecting which results in a final iteration of the original goal. The course had 3 sprints that built on each phase; for example, in sprint one, the students learnt how to collect data from remote application programming interfaces (APIs), filtering the data and presenting only data relevant to the end user in a flat text file. In sprint two, they learned how to build their own simple API so that users could request data, such as had been generated in the previous sprint, and have the data returned in standard computational formats such as JSON and XML, which can be read directly into Python and used in clinical analysis pipelines. In sprint three, the students were asked to reflect on user stories from practice they had submitted earlier in the course and reflections from the work involved in the previous sprints to inspire the focus of their own coding projects, which would be submitted as summative assessment.

The scenario for sprints one and two were delivered in a Jupyter notebook. The unit lead, acting as the product owner, also wrote a preliminary set of requirements in Git Issues. The real-world problem–based learning was sourced from user stories, captured from real-life requests from clinical laboratories to develop the VariantValidator software. The requests were developed into a set of requirements, which acted as the driver for the programming activities, delivered by the product owner. To emulate a real-world scenario, the product owner iteratively changed these requirements throughout the life cycle of the programming project; students therefore had to respond to these changes in an iterative manner and update their code. By using these agile methods to drive and structure the learning, we could provide an immersive environment underpinned with real-world programming problems. The phases of the sprint also fitted neatly with the 3 self-regulated learning phases (forethought, performance control, and self-reflection) to help develop the self-efficacy of the students [13].

### Rule 8: Simulate a community of practice (CoP)

We tried to instil the principles of a community of practice (CoP) into the unit. A CoP is a professional network that has established best practice, a collective ethos or culture and a need to share knowledge and obtain support [20]. Encouraging a CoP in clinical bioinformatics education through the use of problem-based learning has been something that we have been pursuing as an institution for some time [9]. We wanted our students to benefit from establishing a network, not only during the course but afterwards, when they could draw on their collective knowledge and expertise.

We found that the group-based peer learning really did support the students because it gave them an informal environment where they were comfortable expressing any issues and knew they would receive support from their peers. The challenge was to provide students with sufficient support to help deal with any issues they had and draw on the group work for support while also having the autonomy to develop their individual problem-solving skills. This balancing act required significant resource both at the design stage, requiring additional materials for different learner requirements, and during the delivery stage. During delivery, 2 facilitators supporting the lecturers was sufficient to support around 30 students.

Group-based learning was optimised by embedding the educators directly into the CoP within Slack, which meant that they could deal with technical issues arising in an asynchronous fashion while fitting this around their own work commitments. Once issues had been resolved, students were encouraged to feed back to the CoP. The educators also joined in with the discussions ensuring an approachable demeanour. The students enjoyed these interactions and were happy to engage with the educators, which facilitated effective social learning. The unit lead and tutor also benefited from an opportunity to learn lessons from the facilitators (such as technical knowledge), which could be incorporated into the design of the next run; hence, the facilitators formed an active part of the community rather than steering discussions.

In terms of design, we ensured that more individual role-based activities were included within the group work so students still had responsibilities they needed to fulfil as part of the team. The students worked together in teams acting in various roles such as:

- project lead;
- programmers; and
- testers.

Each student performed one of these roles for each sprint with some trying all three. The project overview was given to the students, and they began to work collaboratively to produce a product based on a real-world scenario. As expected, the students began to construct a programme based on this initial outline. The interactive social media environment allowed the unit lead to act as the “product owner” and change the brief dynamically, introducing the concepts of user-led agile development. We believe that this informal interaction with the students was a key reason for the success of the unit.

### Rule 9: Try a team-teaching approach

Team teaching has been known to energise staff and encourage improvements through sharing ideas and drawing on different strengths as well as helping distribute work load across the team [21]. The development team came from an interdisciplinary hub in the School of Health Sciences in the Faculty of Biology, Medicine, and Health at The University of Manchester, which was composed of people from a variety of backgrounds and specialisms [22]. The unit lead is from a research background in genomics and bioinformatics, the unit tutor is from technology-enhanced teaching and learning background, whereas the facilitators have been students from a range of data science disciplines. This mix of skills, backgrounds, and expertise contributed to the creativity in design and development of the materials, which included both the design methods and the materials themselves. For example, instead of dry text–based learning content to introduce the first coding activity, the team recorded a Zoom interview with a clinical bioinformatician instead. Another example of having a multidisciplinary perspective can be seen in the assessment strategy of the course, which encouraged both assessment “of” and “for” learning. Formative assessment was integral within the group work, both through the discussions the learners were having with their peers during the sprints and within the reflections post-sprint. The summative assessment then built on this activity in 3 ways:

- assessed discussion (20%) during the 10 weeks of the course;
- coding project (30%) submitted at the end of the course building on the final sprint; and
- reflective presentation (50%) submitted at the end of the course building on the sprint reflections.

You can see that through the weightings that the coding was not the most important skill set they needed to leave with. This decision was a product of our team-teaching approach, which helped to create innovative materials to capture the interest of a diverse student audience.

An additional benefit of instilling CoP-driven teaching has been that the students have taken ownership of the evolution of the unit. By working in teams, students were able to identify areas of the teaching where the course material could be improved or additional material could be provided to support their learning. To facilitate their ongoing learning, the students wanted to be involved in the development of new material so we have established a cocreation group to generate the material for the next run of the unit. This enables us to provide an environment where students can develop teaching skills, which contribute to their ongoing development since training is also a key aspect of a clinical bioinformatician’s role. We have also invited the developer of another key clinical genomics tool, Leiden Open Variation Database (LOVD) [23], to provide additional user stories so sprints can be updated to reflect the ever-changing clinical genomics landscape. This provides teachers and students with the chance to interact with the developers of tools used in clinical labs and the exchange of ideas informs software development to meet the community need as well as inspiring updated teaching material.

### Rule 10: Facilitate the journey … well!

The role of peer-facilitators, who have recently been through some form of bioinformatics education, has been shown to be effective in the delivery of bioinformatics education to illustrate the application of the discipline in practice, while also developing the mentoring and teaching skills of the facilitators [8]. The courses benefited significantly from having knowledgeable, well-organised, and helpful graduate student facilitators [5]. The facilitators were available at a set time each week so students could ask questions and explore solutions with them. They also flagged any struggling students to the educators so additional support could be devoted to those who needed it. We trained our facilitators by asking them to test the materials, running a training session to set expectations, and giving them a facilitation guide that included hints and tips and models for facilitation including Salmon’s E-moderating Framework [8]. This model involves 5 stages starting with ensuring access and comfort with the environment, building socialisation and information sharing with knowledge building, and ending with development at the end of the process.

Another lesson learned from delivering the course has been the importance of giving clear instructional content to support the learning journey. The course materials needed to provide clear instructions, relevant signposting of resources, and guidance support, importantly before coding begins to ensure that the students are comfortable with the materials. Delivery was then facilitated by less formal interaction with the students using Slack, which encouraged engagement and peer-to-peer conversation and support.

## Discussion

The aim of the Introduction to Programming course was to simulate a real-world experience by building a situated learning environment, within which students could learn current best practices for programming in clinical bioinformatics. This was provided in a safe learning environment providing the learners the space to fail and learn from their mistakes both individually and as a team. Being immersed in this environment facilitated the codevelopment of code, team-based discussion, and problem solving. The results of this original aim can be seen from the 18 responses to the end of course surveys where the majority of students felt they had the basics and were now looking forward to applying their skills further. In terms of Jupyter Notebooks, the students recommended their use and liked how they built their understanding and brought the programming concepts to life. The sprints and team-based working was a highlight for nearly all the students, and the use of Slack was a resounding success, which is reflected in the number of messages and resources shared.

From the results of the assessed discussion activities, it was evident that the students were supporting each others’ learning, which helped instil principles of self-regulated learning to advance their programming and develop problem-solving skills critical to effective future practice. This learning ethos ensured that the students were well positioned to progress as programmers through interacting with peers and sourcing code from community platforms such as Stack Overflow [17]. Experienced facilitators were available asynchronously on Slack throughout the course as well as synchronously at predefined intervals. This enabled agile and adaptive support for the entire cohort, giving the educators the time to focus on supporting those who were struggling.

## Conclusions

In order to continually improve the course, the cocreation project with students and multidisciplinary experts from industry and higher education has recently begun. It will focus on collating more practice-based user stories to encourage problem-solving skills and also have an immediate use and impact on practice. Jupyter Notebooks are increasingly being used in teaching, and the next step is to create a notebook wizard that will support other educators to create Jupyter Notebooks for their teaching. This will help to ensure consistency, quality, and reusability of the notebooks and also support other academic staff in building their awareness and understanding of their application for computational skills-based learning.
